# Psychological needs of critical care staff and barriers to accessing support: A qualitative study

**DOI:** 10.1111/nhs.12958

**Published:** 2022-06-14

**Authors:** Olivia Rae Sutton, Elisabeth Anne Norton

**Affiliations:** ^1^ Clinical Psychology Department University of East Anglia, Norwich Research Park Norfolk UK; ^2^ Norfolk and Norwich University Hospitals NHS Foundation Trust Norfolk UK

**Keywords:** barriers, critical care, critical care staff, mental health, psychology, support

## Abstract

Worldwide, critical care staff are vulnerable to mental health difficulties. Support is varied and uptake is minimal.Therefore, barriers need to be understood in order to be addressed; doing so may improve staff's mental health, resulting in positive consequences. This qualitative research took place between September 2020–November 2020 at a National Health Service critical care unit in England. Participants were critical care staff (*n* = 9). Data were collected through semistructured interviews and analyzed using thematic analysis. The Consolidated Criteria for Reporting Qualitative Studies (COREQ) was used to report the findings, with analysis resulting in six themes: support is the team together in the moment, keeping work‐related difficulties from the forefront of the mind, it's just part of the job, stigma makes it hard to speak up about psychological difficulties, normalizing psychological support, and desire for psychological support within critical care. Psychologist presence in critical care, as well as further options for support, may help to reduce barriers and improve staff mental health. Further research is needed to evaluate staff outcomes across multiple sites to refine understanding and interventional approach.

**Key points**

- Critical care staff would like further options for work‐based psychological support.
- There are barriers for critical care staff to accessing psychological support, including preferring to speak to colleagues, blocking out psychological distress, rationalizing stress and trauma as a normal part of the role, stigma, unfamiliarity, and accessibility.
- Possible methods for improving staff mental health are discussed, including psychological support and other strategies.

## 1 INTRODUCTION

Critical care staff (CCS) are likely to experience burnout, posttraumatic stress disorder (PTSD) symptoms, and other mental health problems due to the demanding and often traumatic nature of their work (e.g., Chuang et al., 2016; Colville et al., 2017; Friganović et al., 2019). The evidence shows this is a global problem, which has had negative consequences for staff retention rates (Khan et al., 2018) and impaired quality of patient care, for example through compassion fatigue and patient mortality rates (Bogue and Bogue, 2020; Schlak et al., 2021; Van Mol et al., 2015). Given the international prevalence of these issues, research has focused on determining individual and organizational risk factors, which have included increased workload, lack of control, and breakdown in work community (e.g., Moss et al., 2016). These are important elements to consider in responding to burnout and mental health problems in CCS; however, proactive management of psychological issues may enhance outcomes. Further research on the individual experiences of CCS that contribute to etiology of psychological difficulties is needed to aid understanding and to develop and implement preventative measures (Mealer et al., 2017).

Much of the literature has explored clinicians' personal ability to manage adverse events, in terms of resilience. Increased resilience through strategies including mindfulness training, mentoring, exercise, counseling, social events, and appropriate breaks have been shown to improve stress management and reduce symptoms of mental health problems (Brown et al., 2018). Promoting resilience is useful for CCS; however, the limitations of this include placing individual responsibility on staff to manage with limited resource or in poor conditions and deter from systemic change within healthcare settings (Baid, 2018; Traynor, 2017). Of note are the strategies that place less responsibility with the individual (such as mentoring, counseling, and social events). Additional factors that have been found to reduce mental health difficulties in CCS reflect this, such as supportive work environments, speaking to seniors, debriefing, and seeking social support (Alharbi et al., 2019; Colville et al., 2017; Schlak et al., 2021).

In the United Kingdom, The Faculty of Intensive Care Medicine and Intensive Care Society ([FICM and ICS] 2019) outline standards for staff well‐being, acknowledging this as a new chapter within the guidance and highlighting growing recognition of staff well‐being. They recommend CCS *should* have access to independent psychological support, although this is not mandatory, suggesting availability may vary across units. In June 2020 19% of critical care units in the United Kingdom reported having access to a psychologist in their unit (ICS, 2020a) and this figure may have been lower before the COVID‐19 pandemic, the impact of which highlighted the longstanding need for psychological support in hospitals (e.g., guidance from the British Psychological Society, Highfield et al., 2020). However, engagement with formal psychological intervention is varied across hospitals; Muller et al. (2020) found that healthcare workers were more likely to access practical and social support rather than professional psychological help. In addition, despite high self‐reported psychological distress only 9% of 913 healthcare workers used psychology services, and 0.9% used the National Health Service (NHS) hotline during acute phases of COVID‐19 (Petrella et al., 2021). The reasons for the disparity between distress, burnout, sickness rates, and uptake of support need to be further understood and addressed.

Moss et al. (2016) propose that a multifaceted approach may be used to target prevention of poor mental health in CCS, including enhancing the environment and intervention to help CCS cope. There are existing positive strategies used at an individual and organizational level, and it is hoped that these may be highlighted and emphasized, to be widely applicable. Furthermore, given recent evidence demonstrating benefits of psychological intervention in healthcare staff (e.g., Barrett and Stewart, 2020; Wade et al., 2020), the authors consider that a psychology team based in critical care would add a further element to improving CCS's mental health.

### 1.1 Aim

This study aims to understand the psychological needs of CCS in a single adult critical care unit and the reasons why provided support is not widely accessed. FICM and ICS (2019) state, “Many of these standards and recommendations are not evidenced directly from patient trials, but are identified from qualitative research studies, governmental or other national agency reports” (p. 103). It is hoped that this research will provide insight to inform developments so that support is available and sought appropriately.

## 2 METHODS

### 2.1 Design

It has been suggested that qualitative research would provide better insight into this area but is lacking within the literature (Friganović et al., 2019). Therefore, a qualitative design was used, conducted through individual semistructured interviews. Findings are reported according to the consolidated criteria for reporting qualitative research (Tong et al., 2007). The research was approved by Health Research Authority and Health and Care Research Wales (REC number: 20/HRA/4282).

### 2.2 Participants and setting

Participants were recruited through purposive sampling from a single adult critical care unit in a hospital in England. The unit held 20 beds, for both high dependency and intensive therapy patients. At the time of the research, two psychologists were present on the unit on a temporary basis for patient care but were not employed to support staff (furthermore, these psychologists are not part of the authorship team). Formal staff support was via occupational health (which required a referral and did not have access to a psychologist).

Inclusion criteria were that participants currently worked in critical care. Participants were excluded if they had not worked in critical care for at least 6 months and during the first wave of COVID‐19, if they were not a fluent English speaker and if they were receiving care for a preexisting mental health difficulty related to factors outside of their job role (this was to ensure interviews captured information relevant to experiences of the role alone). The recruitment period lasted 2 months and 16 CCS responded, nine of whom met the inclusion criteria, which was deemed satisfactory for thematic analysis (Braun & Clarke, 2013). The other seven did not meet criteria for duration worked in critical care or did not deem themselves a fluent English speaker.

All staff within this study worked on the unit on a permanent basis. The nonclinical staff are always present on the unit and are immersed in the difficulties of the environment, often being involved in distressing situations. In addition, research shows that nonclinical staff are just as likely to experience burnout in healthcare settings (Ashill & Rod, 2011; Pindar et al., 2012). Therefore, it was important to take their perspectives into account.

### 2.3 Qualitative data collection

Data collection took place between September and November 2020. Participants completed written consent forms and could withdraw at any time. The researchers did not work on the unit and had no prior relationship with participants. Participants were not offered compensation for taking part and they were made aware of the researchers' job roles and interests in the research topic. Interviews were conducted by the first author either in person (at the hospital) or via video‐call, each lasting between 30 and 45 minutes. No one else was present during interviews and participation was discreet and confidential (e.g., to minimize the possibility that colleagues would know they had taken part). The topic guide was peer reviewed by a critical care consultant and former critical care nurse (Appendix A).

Demographic information was gathered at the beginning of each interview. The length of time participants had worked in critical care ranged from 4.75 years to 20 years. Participants included four nurses (various ranks), a medical consultant, a physiotherapist, an emergency practitioner, and two nonclinical staff. Four were male and five were female. Participants' ages ranged from 31 to 58 years old.

### 2.4 Qualitative data analysis

The authors were a clinical psychologist (BSc, ClinPsyD) and an assistant psychologist (BSc), meaning their motivations were not impartial. Rigor came from keeping a reflective journal about the interviews and positionality and these topics were discussed between authors throughout. Data were rich and similar perspectives emerged in the interviews with reduced variation, suggesting data saturation was reached (Morse, 1995).

Data were analyzed using thematic analysis (Braun & Clarke, 2006). No participants withdrew and therefore all nine interviews were analyzed. Interviews were audio recorded and transcribed verbatim but anonymized. Transcripts were not returned to participants for comment or correction. In line with the authors' reflexivity, an inductive approach was used whereby semantic themes were grounded in the data rather than formed by a theoretical lens (Braun & Clarke, 2006). The authors familiarized themselves with the data set by reading and rereading the transcripts, immersing themselves in the data. Transcripts were then coded line by line for relevance to the research question, findings were discussed, and transcripts were recoded as necessary. Codes were arranged into themes and these were reviewed and deliberated by the authors. Agreement was reached and six main themes were named. Participants were invited to review the initial thematic analysis, but none responded. The researchers reviewed their analysis in line with criteria for trustworthiness of thematic analysis set out by Nowell et al. (2017), in terms of credibility, dependability, and confirmability.

## 3 RESULTS

Six themes relating to psychological experiences and barriers to accessing support were derived from the data.

### 3.1 Support is the team together in the moment

All staff members discussed the importance of having their colleagues around them as consistent figures of support. There was a consensus that the team provided morale and a mutual understanding that was otherwise lacking.

*“I don't think I was very well supported. Um, I mean apart from my colleagues at work who I could talk to …//… and I think we all tried to keep ourselves uplifted”* *(Participant 8)*

*“There isn't really that much of an outlet, you can kind of on your shift ask people how they're doing and if they need to talk about anything… but there's nothing really formally set up for people”* *(Participant 5)*

*“It was mainly the team, I think generally we didn't feel that supported. I certainly didn't.”* *(Participant 6)*

Some staff members found formal avenues of support less accessible, due to lengthy or inconvenient referral processes. Having immediate support from colleagues was a more convenient and instinctive option.

*“If I wanted to speak to a psychologist at the moment, I'd probably have to refer myself …//… and eventually find somebody that could chat to me at some point in the next two or three weeks, or months… it doesn't really tick the box for me, now’” (Participant 4)*.

*“People say ‘if you've got any questions you can write to health and well‐being, and they can support staff’ but that's actually not what support is. Support is the team looking out for each other, in the moment really” (Participant 4).*

### 3.2 Keeping work‐related psychological difficulties from the forefront of the mind

Over half of the staff members spoke about purposefully blocking out difficult psychological experiences both in the moment and for sustained periods to cope long term. All but two of the participants spoke about separating their work and home life as a necessary element of coping when regularly faced with upsetting circumstances at work.

*“We try to block it out as much as possible, we had a patient and you could hear that they were very poorly and that's unpleasant, but you kind of just try and put that to one side, y'know, go to the back of your mind” (Participant 3)*.

*“It's almost like, I come into work and this is like a double life. I have a work life and I have a home life, and I try and keep them quite separate” (Participant 2)*.

*“I think if you take it home with you and don't switch off, that's when it becomes a stress” (Participant 9)*.

Staff members also spoke about using their journey home as a transition period to psychologically compartmentalize the difficulties faced within their shift.

*“Even just driving home from work can be that transition, slowly leaving it behind …//…if I've had a bad day I've always cried in my car and then by the time I'm home, I've done it. It's gone” (Participant 9)*.

*“I live [number] miles away and by the time I get home, this place doesn't exist anymore.” (Participant 2)*.

### 3.3 It's just part of the job

The staff members identified stress and trauma involved in their roles but created a narrative that accepted this as a natural part of working in critical care and enabled them to justify the difficult shifts and carry on with their work.

*“I really wish that there was a way I could eliminate having to deal with end of life …//… I hate that side of the job and yeah it's just part of it sadly” (Participant 8)*.

*“I was just looking at this lad in the bed thinking y'know ‘that could be my son’…//… it's difficult, you just keep going really” (Participant 6)*.

*“I mostly just try to rationalise it to be part of it and ‘that was just a shitty day’ and the next day will probably be better” (Participant 5)*.

Some staff acknowledged the drawbacks of this perspective; in having observed their colleagues during difficult circumstances they considered how it might affect their well‐being.

*“They're just going from one bad, horrible thing to the next without actually giving themselves any time to think about it and that's when people get in, perhaps a little bit of bother.” (Participant 4)*.

*“[The nurses] have got a patient who's dying and very upset family coming in …//… and they'll brush it off and not really acknowledge it and I think ‘that is just the job, but that doesn't mean it's not, hard’” (Participant 1).*

### 3.4 Stigma makes it hard to speak up about psychological difficulties

Seven out of the nine staff members spoke of the stigma associated with experiencing psychological difficulties, particularly within the critical environment they work. There was a sense of needing to appear psychologically robust to be viewed as a reliable member of the team, and the alternative would be a sign of weakness.

*“I think in the unit that I was a part of, um there was this sense that, if you couldn't cope that you were weak.” (Participant 7)*.

*“I think you want to present yourself as a strong character that is able to take on the emotional stress of looking after sick people and people that die as well” (Participant 8)*.

*“You rely on people being strong, in a life and death situation you need to be able to rely on your team and I would feel like I was waving a flag saying ‘you can't rely on me at the moment’” (Participant 1)*.

Similarly, staff members voiced their concerns about being seen to access psychological support because of the culture around strength and coping.

*“Health and well‐being have offered loads of help and it's not been taken up and I think that's a culture thing” (Participant 1)*.

*“I think it would reflect on me as a practitioner in critical care, going to see a psychologist is that ‘they're losing the plot’, is that a sign of weakness? You know ‘oh we need to be a bit careful with [own name] because they're seeing a psychologist’” (Participant 2)*.

As a result of this, several of the staff members highlighted the need for psychological support to be discrete, to avoid others' judgment.

*“I wouldn't want it to be obvious to anybody else, it would need to be away from the unit, very separate, because there is still a huge stigma and I think particularly in this environment” (Participant 1)*.

*“I think I'm a fairly private person, so probably one to one [support would be best]. I don't know that I'm quite so good in a group situation” (Participant 3)*.

### 3.5 Normalizing psychological support

Psychology was spoken of as something unfamiliar and consequently potentially intimidating. All but two of the staff members discussed normalizing psychological support and considered this would encourage and reassure staff to access it when needed.

*“People who maybe haven't come across it or clinical psychology sounds quite terrifying, if they can see that it's really working for the patients then maybe that will make them a little bit more open to it for themselves?” (Participant 1)*.

*“I think that's how you make it most beneficial, you normalise it as something that y'know, the same as I have to MOT my car, I need to MOT myself” (Participant 4)*.

(an MOT is an annual test to check a car meets road safety standards in the United Kingdom)

Seeing their colleagues speaking about personal difficulties with a psychologist on the unit (who was there on a temporary basis for patient care) seemed to reduce shame and allowed them to consider doing the same. CCS also discussed how this made the psychologist more approachable, rather than referring to someone unknown.

*“People will come onto the unit and some of the nurses have gone off to speak to them which I think helps ‘cause it makes you think ‘oh well other people are doing it’ so it normalises it a little bit” (Participant 8)*.

*“You may think you're the only one that thinks something and then by sitting and talking about it, you realise you're not the only one, everybody is feeling a bit like that” (Participant 9)*.

*“I was able to talk to (a psychologist) and seeing them around, it was so much easier to be able to approach them, than not really knowing exactly what that person does normally” (Participant 3)*.

*“There is a (psychologist) for patients and relatives, and I think we can access them for that, as well…//… I think they would be more approachable and people would know who it was. I think that makes a difference” (Participant 5)*.

### 3.6 Desire for psychological support within critical care

Being supported at work was important to all staff interviewed. Most noted the support provided by their family and friends but expressed their desire for a neutral party to share difficulties with.

*“I needed somebody, who wasn't my mum or my partner, or y'know somebody who‐ I didn't wanna burden with these horrific stories that I needed to pick through” (Participant 7)*.

*“It'd be worth talking to somebody, rather than coming home and y'know crying. I think it's good to vent to my partner, but I think also it's probably worth talking to someone who may be able to give me help in another perspective” (Participant 8)*.

*“I do think that it is really important, that staff who are expected to care for others are cared for themselves.” (Participant 7)*.

The staff members shared the opinion that a psychologist who was embedded within the team would be more relatable than an external party, as they would be able to understand staff experiences first hand.

*“I think particularly because [a psychologist in the team] would perhaps know the kind of things that we would be dealing with as well. That makes it easier to, to talk.” (Participant 6)*.

*“If you had somebody internal [to discuss difficulties with] then they'd have a better understanding of the experiences that you're going through.” (Participant 7)*.

*“A psychologist who was a specialist in critical care would have some idea of what we go through.” (Participant 9)*.

All staff members shared this opinion, but two noted the psychologist's role would need to be purely to support staff, as working with them jointly for patients could raise conflicts of interest.

*“Even though it would be work related difficulties, I still feel like there should be that personal, professional barrier there.” (Participant 1)*.

*“Touching on themes that might be very sensitive to you, and then going back into that environment where you're expected to be the person, in charge, or the ‘healthy person’ at least might be a bit of a difficult switch.” (Participant 7)*.

## 4 DISCUSSION

The findings of this study highlight some of the reasons why psychological support is not widely accessed by CCS at this site. The study consolidates research on mental health stigma in healthcare settings (Cohen et al., 2016; Riley et al., 2021) and extends the evidence base of factors that may prevent CCS from seeking formal intervention (Colville et al., 2017; Mealer et al., 2017).

The results showed that some CCS may *consciously* block out difficult emotions for sustained periods of time, which corroborates research showing intensive care staff's preference for avoidant methods of coping (Colville et al., 2014). However, there is ample evidence suggesting emotional avoidance is not sustainable (e.g., Boulanger et al., 2010; Fledderus et al., 2010; Hayes et al., 1996) and instead, it may contribute to burnout and exacerbate PTSD symptoms (Colville et al., 2014; Orcutt et al., 2020). Similarly, not giving time to process the psychological impact of difficult events because “it's just part of the job” risks staff carrying a heavy emotional load. There is a growing evidence base for active processing of emotional events (Rodríguez‐Rey et al., 2019) and education on mental health literacy (Moll et al., 2018) for healthcare staff. This could provide support for psychologically informed reflective practice sessions and psychoeducation to encourage recognition of and opening up to difficult internal experiences to influence help seeking behaviors. Similar existing approaches such as debriefing may be further utilized as this may halve the risk of burnout (Colville et al., 2017). Schwartz rounds are also positive opportunities to bring staff together to reflect and these may be cost‐effective methods to prevent mental ill‐health in CCS.

Stigma is perhaps the most well‐known barrier to help seeking for mental health in healthcare staff (e.g., Cohen et al., 2016; Riley et al., 2021). This may be an unintentional result of certain coping strategies, for example, those who “just keep going” may inadvertently communicate to others that this is the only way to cope and deter others from speaking up (e.g., Moll et al., 2013). The findings show CCS's concerns that others would view them as weak or unable to meet the role's expectations if they were to make mental health problems known. As a result, they stated that if they were to seek support, they would prefer a discreet service away from the unit. However, this directly contrasts with discussions around normalizing psychological support, where participants found it reassuring seeing others speaking to a psychologist on the unit. This contrast suggests psychologist presence in critical care may help to challenge stigma by giving staff opportunity to see they are not alone in their experiences. This could potentially invite a more open culture around mental health and may encourage staff to speak to their seniors about difficulties (a strategy shown to lower distress levels in CCS; Colville et al., 2017). Furthermore, encouraging regular check‐ins may support a proactive mental health culture.

Accounts from all participants showed that their team provided a robust support network, which should not be overlooked. Evidence shows peer support to be correlated with fewer mental health problems in healthcare workers (Muller et al., 2020). Teambuilding and organized social activities may be important elements to consider in the prevention of burnout in CCS, as well as exercise initiatives, walking meetings, and team fundraisers (Coates & Howe, 2014). CCS noted their colleagues' advantage of understanding and empathizing by sharing mutual experiences. However due to the uniqueness of their experiences, there was a perception that outsiders would not be able to understand or provide the desired level of support. This was reflected by participants who considered that it would be easier to discuss difficulties with a psychologist present on the unit because they would have insight into their difficulties. Sharing common ground may improve empathy and allow CCS to speak more honestly about their experiences, rather than forming a therapeutic relationship from scratch. In addition, the findings suggest that uptake of support may increase through accessibility and familiarity if a psychologist were present on the unit. However potential disadvantages may include overfamiliarity and reduced objectivity, which could affect intervention outcomes. Furthermore, some staff highlighted their desire for access to an independent psychologist to avoid conflicts of interest. Alternative and more immediate options for support have been introduced by hospitals worldwide, for example, dedicated spaces and well‐being suites (Rimmer & Chatfield, 2020).

In September–November 2019 annual staff turnover in UK critical care units was reported to be more than 20% and rising (Horsfield, 2020). NHS policy has focused on staff retention (being cheaper and faster than recruiting and training new staff) but despite this focus, retention appears to worsening (Buchan et al., 2019). There are likely several reasons for these rates; however, a systematic review by Khan et al. (2018) highlighted one of the key reasons for nurses leaving critical care was stress and traumatic experiences. The benefit of involving psychology teams within critical care may outweigh the cost of staff sickness rates and burnout; recent research has shown psychological intervention to reduce stress in all domains for intensive care staff (Wade et al., 2020) and significant improvements in stress, burnout, and mental health scores have been shown in healthcare staff post‐psychological intervention (Barrett & Stewart, 2020). Furthermore, Holmberg et al. (2020) found increased psychological flexibility contributed to decreased distress and increased work engagement in intensive care staff.

### 4.1 Limitations

Despite gaining further understanding of the experiences of staff in critical care, this study has limitations. The interviews took place during the COVID‐19 pandemic, which is likely to have affected annual leave, short‐staffing, overtime, and various other stressors. These factors may have influenced participants' accounts and a longitudinal design may help to tailor intervention to the uniqueness of the environment. A small sample size from a single study center was used, and participants' varied roles mean their experiences may have largely differed and influenced data saturation. However, CCS's accounts and experiences were paralleled, and coping strategies were similar across all roles (clinical and nonclinical), suggesting that the critical care environment affects staff in similar ways, regardless of role. Nevertheless, research across multiple sites with a larger sample size may provide richer data, for example, to consider nurses' experiences alone. There are contrasting findings in this study (desire for discreet support versus desire to see others accessing support) that are perhaps representative of a more general paradox in mental health stigma, but this may be explored with further research. Lastly, the research was carried out by a clinical psychology team, which was likely to influence data interpretation; future research would benefit from multidisciplinary analysis. However, as Parker (1999) suggests, qualitative researchers’ positionality, theoretical beliefs, and background inevitably shape the research process.

## 5 RELEVANCE FOR CLINICAL PRACTICE

Based on the findings of this study, stigma, accessibility, and emotional avoidance stand out as key challenges for CCS to overcome in seeking psychological support. Considering these issues, it is important to allow CCS opportunity to acknowledge and manage their psychological health appropriately. In their guidelines, FICM and ICS (2019) state that the standards and recommendations “will require a process of ongoing action and monitoring” (p. 103) suggesting that further improvements can be made to enhance CCS well‐being. This study gives a rationale for further consideration of accessible psychological support within critical care, further opportunity for reflective practice, peer support, and practical strategies. As Moss et al. (2016) suggest, a multifaceted approach is likely to be effective; despite improvements in perceptions of mental health, not everyone will feel comfortable speaking with a psychologist and therefore it is important that several options are available so all staff feel able to preserve their well‐being. However, psychologists are not limited to therapy and may be a valuable resource to critical care at organizational levels. The Workforce Wellbeing Best Practice Framework (The Intensive Care Society, 2020b) and the Guidelines for the Provision of Intensive Care Units (FICM and ICS, 2019) suggest that psychologists can offer leadership consultation and multidisciplinary team working. Further to this, psychologists' skills may be used for further research into the psychological impact of critical care and evaluation of interventional outcomes.

## 6 CONCLUSION

CCS can experience psychological distress and therefore naturally develop coping strategies to respond to this. The findings of this study may be generalisable further than the United Kingdom. They highlight positive existing coping strategies to be built upon and reveal barriers to CCS seeking support for mental health problems at work. In considering these barriers, relevant changes are possible and achievable, and these may improve uptake of support, which could have a positive impact on patient care, staff retention, and prevalence of work‐related psychological difficulties.

## AUTHOR CONTRIBUTIONS

Conception, Design, Analysis, Revision and Final Approvals: ORS and EAN Data collection and Drafting: ORS.

## CONFLICT OF INTEREST

None declared.

## Data Availability

The data that support the findings of this study are available on request from the corresponding author. The data are not publicly available due to privacy or ethical restrictions.

## APPENDIX A Semistructured interview guide

1. What is your current job title and for how long have you worked in critical care?
2. What are aspects of your role which you enjoy?
3. What helps you to feel supported when working in such a demanding area of the hospital?
4. Working in critical care is often seen as a demanding and high stress area, what is your experience of this?
5. Did you feel that work‐based support was better, worse or the same during COVID‐19, and why is this?
6. If you have ever encountered psychological difficulties due to work (such as anxiety, depression, stress, trauma experience) how have you managed these? *(If they have not: do you have any thoughts on how you might manage if you were to encounter psychological difficulties?)*
7. Have you ever taken time off due to work‐related distress?
8. Would you feel comfortable speaking to a psychologist about work‐related psychological difficulties? (Are there barriers to you accessing psychological support?)
9. Would you be more inclined to speak to a psychologist about your well‐being at work, if they were employed specifically to support critical care staff? (Why do you think this is?)
10. What would work‐based psychological support ideally look like for you? (Prompt for: What, e.g. *1:1 or group sessions?* How, e.g. *drop ins or pre‐arranged sessions?* Where, e.g. *externally or on the unit with you?)*

## References

[nhs12958-bib-0001] Alharbi, J. , Jackson, D. , & Usher, K. (2019). Personal characteristics, coping strategies, and resilience impact on compassion fatigue in critical care nurses: A cross‐sectional study. Nursing and Health Sciences, 22, 20–27. 31670474 10.1111/nhs.12650

[nhs12958-bib-0002] Ashill, N. J. , & Rod, M. (2011). Burnout processes in non‐clinical health service encounters. Journal of Business Research, 64, 1116–1127.

[nhs12958-bib-0003] Baid, H. (2018). Resilience in critical care nurses‐ is it desirable? Nursing in Critical Care, 23, 281–282. 30311354 10.1111/nicc.12390

[nhs12958-bib-0004] Barrett, K. , & Stewart, I. (2020). A preliminary comparison of the efficacy of online acceptance and commitment therapy (ACT) and cognitive Behavioural therapy (CBT) stress management interventions for social and healthcare workers. Health and Social Care Community, 29, 113–126. 10.1111/hsc.13074 32613644

[nhs12958-bib-0005] Bogue, T. L. , & Bogue, R. L. (2020). Extinguish burnout in critical care nursing. Critical Care Nursing Clinics, 32, 451–463. 32773185 10.1016/j.cnc.2020.05.007

[nhs12958-bib-0006] Boulanger, J. L. , Hayes, S. C. , & Pistorello, J. (2010). Experiential avoidance as a functional contextual concept. In A. M. Kring & D. M. Sloan (Eds.), Emotion regulation and psychopathology: A transdiagnostic approach to etiology and treatment (pp. 107–136). The Guilford Press.

[nhs12958-bib-0007] Braun, V. , & Clarke, V. (2006). Using thematic analysis in psychology. Qualitative Research in Psychology, 3, 77–101.

[nhs12958-bib-0008] Braun, V. , & Clarke, V. (2013). Successful qualitative research (pp. 50–56). Sage.

[nhs12958-bib-0009] Brown, S. , Whichello, R. , & Price, S. (2018). The impact of resiliency on nurse burnout: An integrative literature review. Medsurg Nursing, 27, 349–378.

[nhs12958-bib-0010] Buchan, A. , Charlesworth, A. , Gershlick, B. , & Seccombe, I. (2019). A critical moment: NHS staffing trends, Retention and Attrition. The Health Foundation Retrieved from https://www.health.org.uk/sites/default/files/upload/publications/2019/A%20Critical%20Moment_1.pdf

[nhs12958-bib-0011] Chuang, C. , Tseng, P. , Lin, C. , Lin, K. , & Chen, Y. (2016). Burnout in the intensive care unit professionals. Medicine, 95, 5629. 10.1097/MD.0000000000005629 PMC5268051 27977605

[nhs12958-bib-0012] Coates, D. D. , & Howe, D. (2014). The design and development of staff wellbeing initiatives: Staff stressors, burnout and emotional exhaustion at children and young People's mental health in Australia. Administration and Policy in Mental Health and Mental Health Services Research, 42, 655–663. 10.1007/s10488-014-0599-4 25307317

[nhs12958-bib-0013] Cohen, D. , Winstanley, S. J. , & Greene, G. (2016). Understanding doctors' attitudes towards self‐disclosure of mental ill health. Occupational Medicine, 66, 383–389. 27030052 10.1093/occmed/kqw024 PMC4913366

[nhs12958-bib-0014] Colville, G. , Dalia, C. , Brierley, J. , Abbas, K. , Morgan, H. , & Perkins‐Porras, L. (2014). Burnout and traumatic stress in staff working in paediatric intensive care: Associations with resilience and coping strategies. Intensive Care Medicine, 41, 364–365. 25403756 10.1007/s00134-014-3559-2

[nhs12958-bib-0015] Colville, G. A. , Smith, J. G. , Brierley, J. , Citron, K. , Nguru, N. M. , Shaunak, P. D. , Tam, O. , & Perkins‐Porras, L. (2017). Coping with staff burnout and work‐related posttraumatic stress in intensive care. Pediatric Critical Care Medicine, 18, 267–273. 10.1097/PCC.0000000000001179 28459762

[nhs12958-bib-0016] Fledderus, M. , Bohlmeijer, E. T. , & Pieterse, M. E. (2010). Does experiential avoidance mediate the effects of maladaptive coping styles on psychopathology and mental health? Behavior Modification, 34, 503–520. 20660354 10.1177/0145445510378379

[nhs12958-bib-0017] Friganović, A. , Selić, P. , & Ilić, B. (2019). Stress and burnout syndrome and their associations with coping and job satisfaction in critical care nurses: A literature review. Psychiatria Danubina, 31, 21–31. 30946714

[nhs12958-bib-0018] Hayes, S. C. , Wilson, K. G. , Gifford, E. V. , Follette, V. M. , & Strosahl, K. (1996). Experiential avoidance and behavioral disorders: A functional dimensional approach to diagnosis and treatment. Journal of Consulting and Clinical Psychology, 64, 1152–1168. 8991302 10.1037//0022-006x.64.6.1152

[nhs12958-bib-0019] Highfield, J. , Johnston, E. , Jones, T. , Kinman, G. , Maunder, R. , Monaghan, L. , et al. (2020). The psychological needs of healthcare staff as a result of the coronavirus pandemic (pp. 1–6). The British Psychological Society.

[nhs12958-bib-0020] Holmberg, J. , Kemani, M. K. , Holmström, L. , Lars‐Göran, Ö. , & Wicksell, R. K. (2020). Psychological flexibility and its relationship to distress and work engagement among intensive care medical staff. Frontiers in Psychology, 11, 3010. 10.3389/fpsyg.2020.603986 PMC7672021 33250832

[nhs12958-bib-0021] Horsfield, C. (2020). National Critical Care Nursing Workforce Survey overview report. Critical Care Networks‐ National Nurse Leads (CC3N) Retrieved from https://www.cc3n.org.uk/uploads/9/8/4/2/98425184/national_critical_care_nursing_workforce_survey_report_july_2020_final_v.._.pdf

[nhs12958-bib-0022] Khan, N. , Jackson, D. , Stayt, L. , & Walthall, H. (2018). Factors influencing nurses' intentions to leave adult critical care settings. Nursing in Critical Care, 24, 24–32. 29635820 10.1111/nicc.12348

[nhs12958-bib-0023] Mealer, M. , Jones, J. , & Meek, P. (2017). Factors affecting resilience and development of posttraumatic stress disorder in critical care nurses. American Journal of Critical Care, 26, 184–192. 28461539 10.4037/ajcc2017798 PMC5685839

[nhs12958-bib-0024] Moll, S. , Eakin, J. , Franche, R. L. , & Strike, C. (2013). When health care workers experience mental ill health: Institutional practices of silence. Qualitative Health Research, 23, 167–179. 23132130 10.1177/1049732312466296

[nhs12958-bib-0025] Moll, S. E. , Patten, S. , Stuart, H. , MacDermid, J. C. , & Kirsh, B. (2018). Beyond silence: A randomized, parallel‐group trial exploring the impact of workplace mental health literacy training with healthcare employees. The Canadian Journal of Psychiatry, 63, 826–833. 29673271 10.1177/0706743718766051 PMC6309037

[nhs12958-bib-0026] Morse, J. M. (1995). The significance of saturation. Qualitative Health Research, 5, 147–149.

[nhs12958-bib-0027] Moss, M. , Good, V. S. , Gozal, D. , Kleinpell, R. , & Sessler, C. N. (2016). An official critical care societies collaborative statement‐ burnout syndrome in critical care health‐care professionals. A Call for Action. Chest, 150, 17–26. 10.1016/j.chest.2016.02.649 27396776

[nhs12958-bib-0028] Muller, A. E. , Hafstad, E. V. , Himmels, J. P. , Smedslund, G. , Flottorp, S. , Stensland, S. Ø. , Stroobants, S. , Van de Velde, S. , & Vist, E. G. (2020). The mental health impact of the COVID‐19 pandemic on healthcare workers, and interventions to help them: A rapid systematic review. Psychiatry Research, 293, 113441. 32898840 10.1016/j.psychres.2020.113441 PMC7462563

[nhs12958-bib-0029] Nowell, L. S. , Norris, J. M. , White, D. E. , & Moules, N. J. (2017). Thematic analysis: Striving to meet the trustworthiness criteria. International Journal of Qualitative Methods, 16, 1–13.

[nhs12958-bib-0030] Orcutt, H. K. , Reffi, A. N. , & Ellis, R. A. (2020). Experiential avoidance and PTSD. In M. T. Tull & N. A. Kimbrel (Eds.), Emotion in posttraumatic stress disorder (pp. 409–436). Academic Press. 10.1016/B978-0-12-816022-0.00014-4

[nhs12958-bib-0031] Parker, I. (1999). Qualitative data and the subjectivity of ‘objective’ facts. In D. Dorling & L. Simpson (Eds.), Statistics in society: The arithmetic of politics (pp. 83–88). Arnold.

[nhs12958-bib-0032] Petrella, A. R. , Hughes, L. , Fern, L. A. , Monaghan, L. , Hannon, B. , Waters, A. , & Taylor, R. M. (2021). Healthcare staff well‐being and use of support services during COVID‐19: A UKperspective. General Psychiatry, 34, e100458. 34222795 10.1136/gpsych-2020-100458 PMC8228653

[nhs12958-bib-0033] Pindar, S. K. , Coker, A. O. , Wakil, M. A. , & Morakinyo, O. (2012). Comparison of burnout syndrome among clinical and non‐clinical staff of two tertiary health institutions in Maiduguri, Nigeria. Transnational Journal of Science and Technology, 2, 57–73.

[nhs12958-bib-0034] Riley, R. , Buszewicz, M. , Kokab, F. , Teoh, K. , Gopfert, A. , Taylor, A. K. , Van Hove, M. , Martin, J. , Appleby, L. , & Chew‐Graham, C. (2021). Sources of work‐related psychological distress experienced by UK‐wide foundation and junior doctors: A qualitative study. BMJ Open, 11, e043521. 10.1136/bmjopen-2020-043521 PMC8231022 34162634

[nhs12958-bib-0035] Rimmer, A. , & Chatfield, C. (2020). What organisations around the world are doing to help improve doctors' wellbeing. British Medical Journal, 369, m1541. 10.1136/bmj.m1541 32303490

[nhs12958-bib-0036] Rodríguez‐Rey, R. , Palacios, A. , Alonso‐Tapia, J. , Pérez, E. , Álvarez, E. , Coca, A. , Mencía, S. , Marcos, A. , Mayordormo‐Colunga, J. , Fernández, F. , Gómez, F. , Cruz, J. , Ordóñez, O. , & Llorente, A. (2019). Burnout and posttraumatic stress in paediatric critical care personnel: Prediction from resilience and coping styles. Australian Critical Care, 32, 46–53. 29605169 10.1016/j.aucc.2018.02.003

[nhs12958-bib-0037] Schlak, A. E. , Aiken, L. H. , Chittams, J. , Poghosyan, L. , & McHugh, M. (2021). Leveraging the work environment to minimize the negative impact of nurse burnout on patient outcomes. Environmental Research and Public Health, 18, 610–625. 10.3390/ijerph18020610 PMC7828279 33445764

[nhs12958-bib-0038] The Faculty of Intensive Care Medicine and Intensive Care Society . (2019). *Guidelines for the provision of intensive care services V2* (pp. 102‐104). Retrieved from https://www.ficm.ac.uk/standards-research-revalidation/guidelines-provision-intensive-care-services-v2

[nhs12958-bib-0039] The Intensive Care Society . (2020a). ICS benchmarking of ICU psychology workforce. The Intensive Care Society, 1, 2–3.

[nhs12958-bib-0040] The Intensive Care Society . (2020b). Intensive care as a positive place to work: Workforce wellbeing best practice framework. The Intensive Care Society, 1, 13–15.

[nhs12958-bib-0041] Tong, A. , Sainsbury, P. , & Craig, J. (2007). Consolidated Criteria for Reporting Qualitative Research (COREQ): A 32‐item checklist for interviews and focus groups. International Journal for Quality in Health Care, 19, 349–357. 17872937 10.1093/intqhc/mzm042

[nhs12958-bib-0042] Traynor, M. (2017). Critical resilience for nurses. An evidence‐based guide to survival and change in the modern NHS. Routledge. 10.4324/9781315638928

[nhs12958-bib-0043] Van Mol, M. C. , Kompanje, E. J. , Boit, D. D. , Bakker, J. , & Nijkamp, M. D. (2015). The prevalence of compassion fatigue and burnout among healthcare professionals in intensive care units: A systematic review. PLoS One, 10, e0136955. 26322644 10.1371/journal.pone.0136955 PMC4554995

[nhs12958-bib-0044] Wade, D. , Georgieva, M. , Gunnewicht, H. , Finnigan, J. , & MacCallum, N. (2020). Delivery of a psychological intervention to assess and reduce workplace stress among intensive care staff. Journal of the Intensive Care Society, 22, 52–59. 33643433 10.1177/1751143719884855 PMC7890752

